# A Coast-to-Coast Assessment of Cumulative Impacts and Protection Potential in Canadian Marine Conservation Networks

**DOI:** 10.1007/s00267-026-02427-y

**Published:** 2026-04-16

**Authors:** Grace E. P. Murphy, Selina Agbayani, Jocelyn C. Nelson, Emily M. Rubidge, Ryan R. E. Stanley, Cathryn C. Murray, Noreen E. Kelly

**Affiliations:** 1 Bedford Institute of Oceanography, Fisheries & Oceans Canada, Dartmouth, NS Canada; 2 Institute of Ocean Sciences, Fisheries & Oceans Canada, Sidney, BC Canada; 3 Pacific Biological Station, Fisheries & Oceans Canada, Nanaimo, BC Canada

**Keywords:** Cumulative effects, Human activities, Conservation planning, Marine protected area, Marine conservation network, Scenario analysis

## Abstract

The effectiveness of marine conservation networks depends on their ability to manage and safeguard against the many overlapping human activities and stressors influencing marine ecosystems. Cumulative impact mapping (CIM) combines information on the intensity and location of stressors with the spatial extent and vulnerability of marine habitats to generate a single, relative cumulative impact (CI) score. Despite its potential benefits to inform management and conservation efforts, CIM is not commonly incorporated into conservation network planning. Here, we combine published CIMs from the Canadian Atlantic and Pacific bioregions with existing marine conservation areas (MCAs) to compare impacts inside each MCA against regional impacts. We then conduct a scenario analysis to quantify potential reduction in cumulative impacts in two bioregion’s proposed marine conservation networks by modeling the removal of prohibited activities (bottom contact fishing, dredging, disposal at sea) in alignment with the Canadian Marine Protection Standard. Both regions exhibited similar distributions in current CI scores, with approximately half of MCAs scoring significantly above the regional mean, and the other half below. The scenario analysis predicted a 7% mean decrease (range 0–36%) in cumulative impacts across the Scotian Shelf and 2% mean decrease (range 0–17%) across the Northern Shelf networks. We identified 17 MCAs in the Scotian Shelf and 8 in the Northern Shelf as having high protection potential, which can be a valuable decision support tool for conservation planners. We discuss how CIM can be operationalized for conservation network planning and management.

## Introduction

Marine Protected Areas (MPAs) are spatial designations intended to conserve biodiversity, habitats, and ecological processes by reducing or restricting exploitation and other human activities within defined boundaries. MPAs can be implemented as part of broader networks of protected areas that also include other effective conservation measures (e.g., fisheries closures), collectively referred to as Marine Conservation Areas (MCAs). MCAs restrict varying types of human activities with the aim of improving biodiversity, protecting vulnerable habitats, and preserving ecosystem services (Ban et al. 2019; Edgar et al. 2014). These conservation measures can generate ecosystem benefits and measurable economic contributions, such as enhanced fisheries productivity, tourism opportunities, and climate change mitigation (Costello 2024; Jankowska et al. 2022; Marshall et al. 2019), fostering a resilient blue economy that balances sustainable ocean resource use with the health of marine ecosystems (Mak et al. 2025). When implemented and designed as a network, MCAs collectively ensure that protection is representative of regional ecosystems and safeguards key ecosystem functions (Gaines et al. 2010; Balbar et al. 2020). Globally, marine conservation networks function as spatial management approaches to protect the environment, manage human pressures, restore ecosystems, and contribute to international conservation targets such as the United Nations Global Biodiversity Framework’s (GBF) goal to protect 30% of the ocean by 2030 (CBD 2022). The effectiveness of marine conservation networks will depend on their ability to protect against and enhance resilience to the many overlapping human activities and stressors influencing marine habitats, such as commercial fishing, shipping, deep-sea mining, and aquaculture (Batista et al. 2014; Roberts et al. 2018, Zupan et al. 2018; Sullivan-Stack et al. 2024; Martins et al. 2025). The overlap and interaction of human activities and stressors within MCAs and with exogenous pressures occurring outside the boundaries, like climate change and pollution, introduce the additional management challenge to address cumulative impacts within MCAs (O’Regan et al. 2021; Zentner et al. 2023).

Cumulative impacts, sometimes referred to as cumulative effects, can be defined as the interactions between multiple human activities and natural processes in shared space and time (CCME 2014; Krzyzanowski 2011). Globally, only a fraction of MCA plans mention cumulative effects (34%), and even fewer meaningfully integrate the cumulative effects of multiple human activities into the conservation network design and management process (Murray et al. 2025). Despite significant advances in risk assessment and cumulative impacts methodologies, their outputs are rarely operationalised into conservation planning and decision making, leaving a gap between assessment and application (Murray et al. 2025). Ignoring interacting stressors and the resulting cumulative impacts in conservation planning can lead to underestimated risks to species and habitats and ultimately to ineffective conservation plans (Schultz 2010, Brown et al. 2014; Griffiths et al. 2020; Griffiths et al. 2024). For example, while MCAs often restrict commercial fishing and select marine-based activities (e.g., oil and gas exploration, marine disposal, etc.), cumulative impact analyses have revealed that marine protection measures may still be insufficient if they do not consider the additional pressures imposed by outside factors such as climate change and land-based stressors (Tulloch et al. 2020; Loiseau et al. 2021).

Cumulative impact mapping (CIM) is a well-established spatial modeling method that has been applied to marine regions worldwide to estimate the cumulative effects arising from multiple human activities and stressors (Halpern et al. 2008; Ban et al. 2010; Murray et al. 2015). CIM combines spatial information on the intensity of stressors with the spatial extent of marine habitats and their vulnerabilities to those stressors, into a single, relative cumulative impact (CI) score. Uncertainty and sensitivity of CI scores can be estimated to assess and compare the robustness of results and identify influential factors for model outputs. By quantifying and visualizing the intensity of cumulative impacts, CIM has significant potential to inform management and conservation efforts (Murray et al. 2024), particularly in marine spatial planning (MSP) and conservation network planning (Fernandes et al. 2020; Hammar et al 2020; Willsteed et al 2018; Robb et al. 2024). MSP is an ecosystem-based management approach used to evaluate the spatial overlap of multiple ocean activities to balance ecosystem health and sustainable ocean use (Dominguez-Tejo et al. 2016). CIM has been employed as a decision support tool for MSP initiatives worldwide (da Luz Fernandes et al. 2017; Markantonatou et al. 2021). For example, CIMs have been used to estimate the impact of future human activities (Murray et al. 2015; Hammar et al. 2020), demonstrate how land-based activities contribute to marine cumulative impacts (Loiseau et al. 2021), account for transitory cumulative impacts through ecosystem connectivity (Jonsson et al. 2021), and highlight the importance of transboundary MSP to manage cumulative impacts (Ma et al. 2023). Together MSP and conservation network planning provide complementary mechanisms for managing the distribution and intensity of human influences on marine ecosystems (Portman 2011).

There are clear benefits to incorporating CIM assessments into conservation network planning. CIM provides an analytic framework that clarifies how protection aligns with areas of cumulative impacts and helps identify sites that are highly impacted but also contain habitats targeted for protection. This supports the design, evaluation, and ongoing adaptive management of MCA networks, offering a basis to prioritize protections and to quantify coverage after a network is established. In areas with high cumulative impacts from manageable activities, recovery can be incorporated into design decisions and site prioritization. Conversely, when the focus is conservation of relatively natural or pristine environments, areas with low CI scores can be prioritized (King et al. 2021; Robb et al. 2024). MCAs also often include a spatial management context, in which human activities are regulated in zones to achieve conservation objectives while balancing access to marine areas. CIM provides an evaluation context to understand how well these activities are managed by, or among, sites at the regional (network) scale. Similarly, CIM can support ongoing adaptive management, adjusting protections as cumulative pressures evolve. When impacts are manageable and recovery is feasible, identifying the overlap between CI hotspots and protections can be advantageous; however, when CI scores indicate high impact with minimal habitat types targeted for protection, protections may be better placed elsewhere or paired with targeted management. In short, CIM helps diagnose coverage gaps and informs evidence-based network design and adaptive management.

CIM can also benefit conservation network planning by providing the baseline data necessary for future monitoring. Monitoring MCAs after establishment is challenging and includes monitoring for not only ecological integrity and conservation objectives, but also compliance (Cucinelli 2020; McGeady et al. 2023; Dunham et al. 2024). Current recommendations to repeat CIM exercises every five to ten years to quantify change in cumulative impacts in an area over time (Halpern et al. 2015) can also be valuable as a standardized and cost-effective method to track increasing human pressures within and outside conservation networks and how they may influence conservation outcomes.

Finally, scenario analyses using CIMs can be used to estimate the cumulative impact offsets potentially gained by protecting an area of interest or network of conservation measures. Offset strategies, such as biodiversity or carbon offsets, are an increasingly popular approach in ecosystem management where interventions are used to compensate for the ecological consequences associated with development and ocean use (Thebaud et al. 2015). With human activities and their cumulative impacts in marine regions intensifying (Halpern et al. 2019) and as the global conservation footprint expands (Gurney et al. 2023), there is opportunity to operationalize CIM-informed offsets within MCA networks. Understanding the proportional gains in offsetting local or regional cumulative impacts can inform both the planning and evaluation of MCAs, providing a transparent metric for communicating existing or potential benefits to stakeholders and guiding site-design decisions, such as activity zonation, and how these decisions influence cumulative impacts to protected ecosystems.

Comprehensive CIMs have recently been published for marine bioregions on Canada’s Pacific (Agbayani et al. 2024a) and Atlantic (Murphy et al. 2024) coasts. Both CIMs revealed that each bioregion experienced cumulative impacts from human activities across nearly their full extents, with climate change stressors and commercial fishing being the most pervasive drivers of cumulative impacts on both coasts. Commercial vessel traffic was also a top-five contributor to cumulative impacts in the Pacific, with invasive species among the top-five highest contributors in the Atlantic. While not directly comparable due to differences in activity and stressor data availability and resolution, each bioregion’s CIM was developed following the same methodology and these results provide the opportunity to assess relative cumulative impacts between Canada’s coasts.

With the longest coastline in the world (Lemmen et al. 2016), globally significant fishing grounds (DFO 2021a; Townsend and Pettigrew 1997), numerous rare species and ecosystems (Krautter et al. 2001; Buhl**-**Mortensen et al. 2015; Chu et al. 2019), and significant carbon storage within the seabed (Epstein et al. 2024), Canada’s oceans are among the most productive and diverse marine regions in the world. Marine conservation networks will be key tools for safeguarding Canada’s ecologically and economically important marine ecosystems and the biodiversity they harbor (Schram et al. 2019). Canada has made significant progress in its commitment to meeting marine conservation objectives, protecting 15.5% (~870,000 km^2^) of its marine and coastal areas with MCAs as of fall 2025 (CPCAD 2025). In 2023, the Canadian Marine Protection Standard (CMPS) was established, applicable to all MPAs created since April 2019. This standard outlines activities that are not compatible with the long-term preservation of biodiversity (i.e., oil, gas, and mineral exploration, disposal of waste and other material, and use of bottom-contact fishing gear) and provides consistency regarding the human activities prohibited in Canadian MPAs (DFO 2021b; DFO 2023). The establishment and use of the CMPS sets Canada apart from other countries abiding by the UN Global Biodiversity Framework marine protection targets, many of whom do not employ consistent guidelines about prohibited activities in MPAs (Stanley et al. 2025). Given the recently developed CIMs on both the Atlantic and Pacific coasts, which coincide with conservation network planning in both regions and the ambitious conservation goals underway, Canada provides an ideal case study to assess the influence of cumulative human impacts in MCAs and explore the usefulness of CIM in conservation network planning.

In this paper, we combine recently published CIMs for the Canadian Pacific bioregions (Agbayani et al. 2024a; Agbayani et al. 2024b) and the Scotian Shelf bioregion in Atlantic Canada (Murphy et al. 2024; Murphy and Kelly 2023) with existing federal MCAs (DFO 2024a; CPCAD 2025) to compare cumulative impacts inside each MCA against the full background of regional impacts. We assess the contribution of five sectors (climate, fishing, marine, coastal, and land-based) to CI scores in each of the existing MCAs on both coasts. Using the CMPS as a guide, we also conduct a scenario analysis to quantify the potential CI score reduction associated with the implementation of conservation networks (including both existing and proposed MCAs) in two bioregions (Northern Shelf/Great Bear Sea Bioregion and Scotian Shelf-Bay of Fundy Bioregion), and identify which MCAs are expected to have the largest gain in protection potential for marine habitats. By applying CIM scenario analysis to identify MCAs with high potential for conservation gains through impact mitigation, we demonstrate the usefulness of data-driven tools to aid in decision-making for marine conservation and spatial planners.

## Methods

### Study Areas

**Fig. 1:**
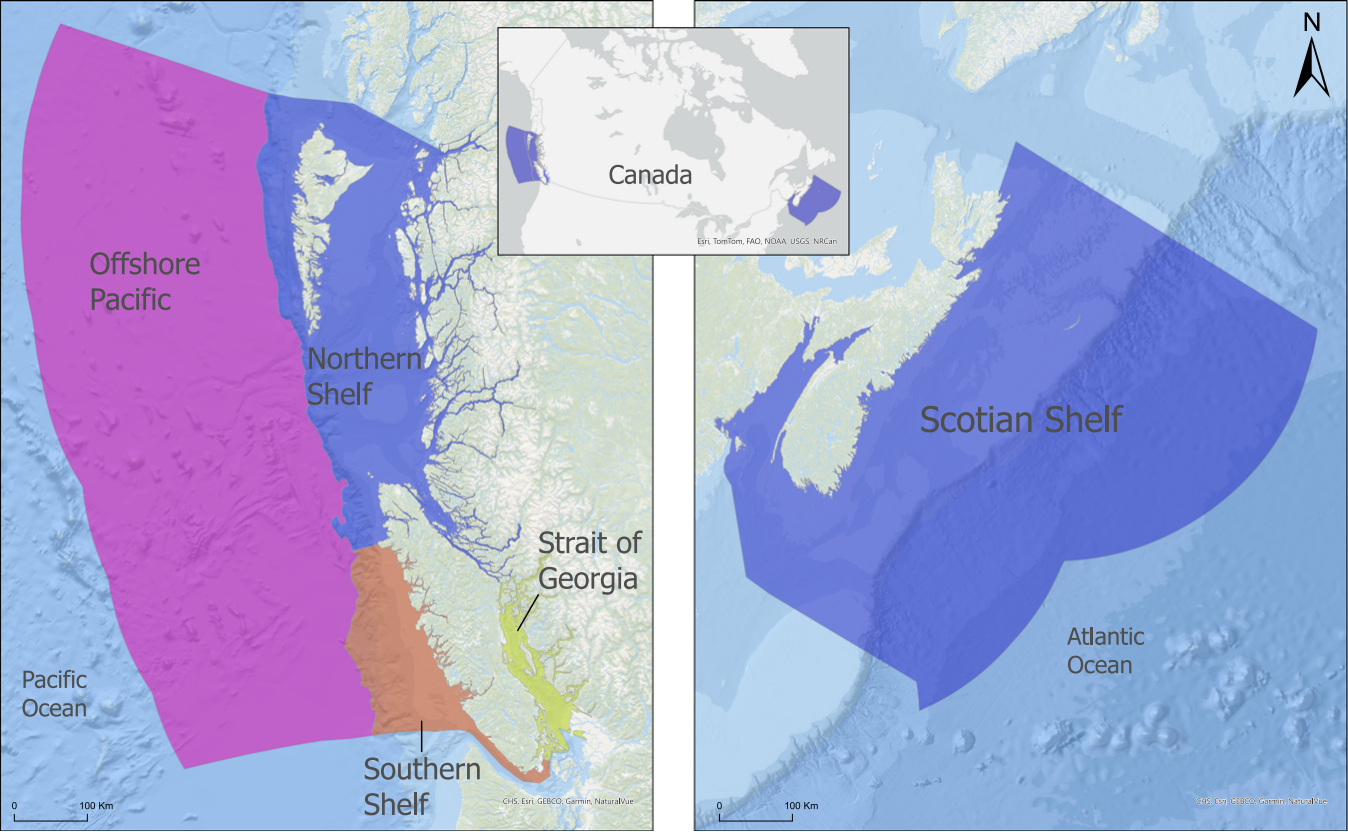
Boundaries of bioregions (four in Pacific Canada and one in Atlantic Canada) included in this study

This study focuses on one bioregion in Atlantic Canada (Scotian Shelf) and all four bioregions in Pacific Canada (Southern Shelf, Northern Shelf, Strait of Georgia, and Offshore Pacific), as designated by Fisheries and Oceans Canada (DFO 2016; Fig. 1). We restricted our eastern analysis to the Scotian Shelf-Bay of Fundy bioregion as CIMs using the same methodology have not yet been developed for the other bioregions and this is the only bioregion in Atlantic Canada undertaking systematic conservation network planning. While the CIMs for the Scotian Shelf and Pacific bioregions used in this paper were created using similar methodologies, variations in the availability and resolution of human activity and habitat data (Tables S1 and S2) prevent direct comparison of the final CI scores between regions. While both regions included activities from the same five sectors (Climate, Commercial Fishing, Marine, Coastal, and Land), in some cases the types of activities included, and the metrics used to represent intensity, differed between the two regions (Table S1). For example, the data used to represent commercial fishing activity in the Scotian Shelf CIM was grouped by gear types, resulting in 15 commercial fishing activity layers included in the Scotian Shelf CIM (Murphy et al. 2024). Commercial fishing data classified by both gear type and species was available for the Pacific region and this allowed the inclusion of 22 commercial fishing activity layers included in the Pacific CIM (Agbayani et al. 2024). Since the CI score calculation is additive, including more activity layers will naturally produce higher CI scores. Therefore, raw CI scores should only be compared within the same region. This paper focuses on comparing relative CI metrics (e.g., low-medium-high cumulative impact classes) rather than raw scores.

The Scotian Shelf-Bay of Fundy bioregion, hereafter referred to as the Scotian Shelf, covers approximately 476,000 km^2^ with 8600 km of coastline and includes both the Scotian Shelf off the coast of Nova Scotia and the Bay of Fundy (Fig. 1). A large variety of habitats and ecosystems exist in the bioregion including coastal bays, extensive mudflats, offshore banks, deep submarine canyons, and biogenic habitats such as seagrass meadows, kelp forests, rockweed beds, saltmarshes, and deep-sea corals and sponges.

There are 4 bioregions in Pacific Canada (Fig. 1), featuring rocky and soft shores, fjords, estuaries, hydrothermal vents, seamounts, bathyal plains, and cold seeps, plus biogenic habitats including seagrass meadows, kelp forests, sponge reefs, and chemosynthetic communities (Jeffery et al. 2023). The Northern Shelf Bioregion (also referred to as the Great Bear Sea) covers approximately 102,000 km^2^, with 29,385 km of coastline (MPA Network 2023), the Southern Shelf Bioregion is approximately 28,000 km^2^, the Strait of Georgia Bioregion is approximately 9000 km^2^, and the Offshore Pacific Bioregion, the largest Pacific bioregion, covers approximately 316,000 km^2^ (DFO 2022).

### Cumulative Impacts in Existing MCAs

The CIMs used in this paper (Agbayani et al. 2024a; Murphy et al. 2024) were originally created at a 1 km^2^ resolution following the methodology developed by Halpern et al. (2008), which combines several types of spatial information. First, the spatial extent and intensity of individual human activities or stressors (hereafter “stressors”) are overlaid with the spatial extent of habitats. Then, an impact score is calculated using a vulnerability rating that estimates the sensitivity of each habitat to each overlapping stressor (Halpern et al. 2008; Murray et al. 2024). The Pacific CIM included 46 stressors and 38 habitat classes. The Scotian Shelf CIM included 45 stressors and 21 habitat classes. The CIM model is additive such that the final CI score is the sum of all stressor–habitat impact scores for each 1 km^2^ grid cell.

To quantify cumulative impacts in each region’s existing marine conservation networks we clipped the regional CIMs (Agbayani et al. 2024b; Murphy and Kelly 2023) to existing MCA boundaries extracted from the Canadian Protected and Conserved Area Database (CPCAD 2025). From the CPCAD, we selected only protected areas in the marine biome covered by federal regulations. For MCAs split into multiple zones (e.g., core protection and adaptive management zones) we merged zones into a single MCA. Since the minimum spatial resolution of both CIMs was calculated on 1 km^2^ grid cells, we excluded MCAs from the analysis with a total area less than 1 km^2^. In the Scotian Shelf bioregion 36 MCA entries from the marine biome were included in the CPCAD. Seven MCAs were excluded from the analysis as they were classified in the CPCAD as only having provincial or private ownership. Three remaining MCAs were excluded as they had an area less than 1 km^2^. After merging the multiple management zones included in some MCAs, 16 existing MCAs in the Scotian Shelf bioregion remained for this analysis. Across the four Pacific bioregions, 204 MCA entries from the marine biome were included in the CPCAD. One hundred seventy-four MCAs were excluded from the analysis as they were classified in the CPCAD as only having provincial or private ownership. We included the Gwa̲xdlala/Nala̲xdlala (Lull/Hoeya) Marine Refuge as it is an Indigenous Protected and Conserved Area and has joint governance. Eleven remaining MCAs were excluded as they had an area less than 1 km^2^. After merging the multiple management zones included in some MCAs, 18 MCAs in the Pacific bioregions remained for this analysis.

We used analysis of variance (ANOVA) to assess whether mean CI scores within existing MCAs significantly differed from mean bioregional CI scores (including MCAs). We classified MCAs for each region as low, medium, and high cumulative impact classes (hereafter referred to as “impact classes”) using Jenks Natural Breaks for CI scores across the entire region. Jenks Natural Breaks is a classification method that identifies natural groupings in numeric data by minimizing variance within classes and maximizing variance between classes such that the impact classes are as distinct as possible (Jenks 1967). For the Scotian Shelf, the low impact class ranged between 0–10.73, medium between 10.74–16.82 and high between 16.83–42.7. For the Pacific bioregions, the low impact class ranged between 2-22.35, medium between 23.35–58.67, and high between 58.67–224.41. We evaluated the current level of protection by comparing total and sector-specific CI scores across the existing MCAs with the full region (inclusive of MCAs) by calculating the percent overlap of the frequency distributions. Spatial analyses were conducted in ArcGIS Pro v.3.3.2 (ESRI 2023). Statistical analyses were conducted in R v.4.3.1 (R Core Team 2022). Percent overlap of frequency distributions was calculated using the “overlapping” package in R (Pastore et al. 2025).

### Cumulative Impact Protection Potential Across Proposed Marine Conservation Networks

We conducted scenario analyses to assess the percent CI score decrease (i.e., protection potential) following the implementation of the Canadian Marine Protection Standards in proposed marine conservation networks in two bioregions: Scotian Shelf on the Atlantic coast and Northern Shelf on the Pacific coast, including both existing conservation measures (available from CPCAD 2025) and proposed conservation sites (Beaty et al. 2024; King et al. 2021). Protection levels have not been established in the proposed sites, therefore we used the CMPS as a guide to estimate potential prohibited activities and remove them from MCAs (DFO 2023). We are assuming the CMPS will be applied for the purposes of this analysis to illustrate how CIM can be used in conservation network planning. MCAs designated by solely provincial or First Nations governments are not directly subject to the same protection standards so we only include federally regulated MCAs in the scenario analysis. The CMPS outlines activities legally prohibited in federal MCAs including oil, gas and mineral exploration, disposal of waste and other material, and use of bottom-contact fishing gear. In each region, we removed the presence and intensity of five activities that fell within these restricted categories from within the boundaries of all existing and proposed MCAs. In the Scotian Shelf, we removed four types of commercial fishing (dredge boat, bottom otter trawl, shrimp trawl, and Danish/Scottish seine) plus disposal at sea activities. In the Northern Shelf, we removed three types of commercial fishing (groundfish bottom trawl, shrimp trawl, and scallop trawl) along with disposal at sea and dredging activities. After removing the activities from within the MCA borders, we re-calculated the total CI scores and evaluated the percentage reduction in CI scores resulting from the removal for each MCA across the conservation network in each region, respectively. We considered MCAs to have high protection potential when both their current CI score and projected CI score decline exceeded the regional medians, indicating areas that are currently heavily impacted by multiple activities and stressors, but have strong capacity for improvement following protection. Scenario analyses were conducted in R v.4.3.1 (R Core Team 2022) using the original CIM outputs from Murphy et al. (2024) and Agbayani et al. (2024a), which provide impact scores for all activities at a 1 km^2^ resolution. From this, we excluded the five restricted activities in each region and re-calculated the CI scores by summing impact scores across all remaining activities.

## Results

### Cumulative Impacts in Existing MCAs

**Fig. 2:**
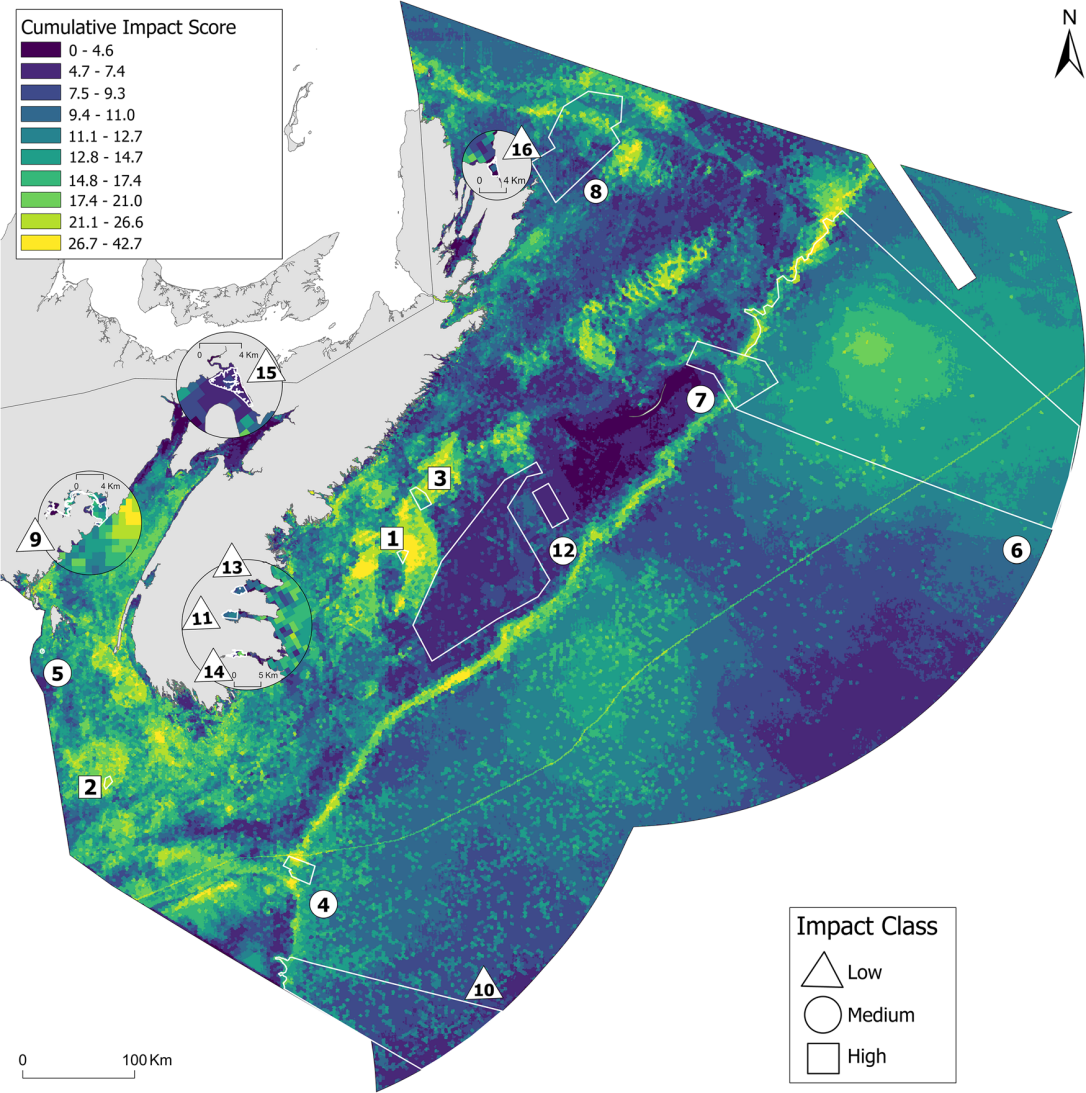
CI scores for existing marine conservation areas (MCAs) (*n* = 16) in the Scotian Shelf bioregion in Atlantic Canada. Numbers rank MCAs from highest mean CI score (#1) to lowest mean CI score (#16), and symbols classify mean CI in each MCA as Low, Medium, or High based on Jenks natural breaks for CI scores across the entire region. See Fig. 3 for corresponding names of each MCA. Circles indicate areas of the coast that are magnified (see individual scale bars)

**Fig. 3:**
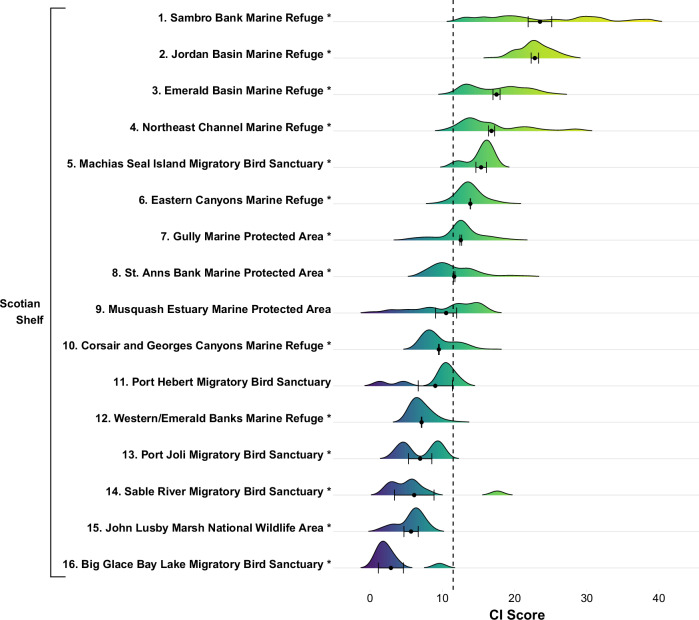
Distribution of CI scores for existing MCAs (*n* = 16) in the Scotian Shelf bioregion in Atlantic Canada. Black circles represent the mean CI score (±95% confidence intervals) within each MCA. Numbers rank MCAs from highest mean CI score (#1) to lowest mean CI score (#16). Asterisks indicate that the mean CI score in an MCA is significantly different (*p* < 0.05) from the mean bioregional CI score, inclusive of existing MCAs (dashed line), according to ANOVA

**Fig. 4:**
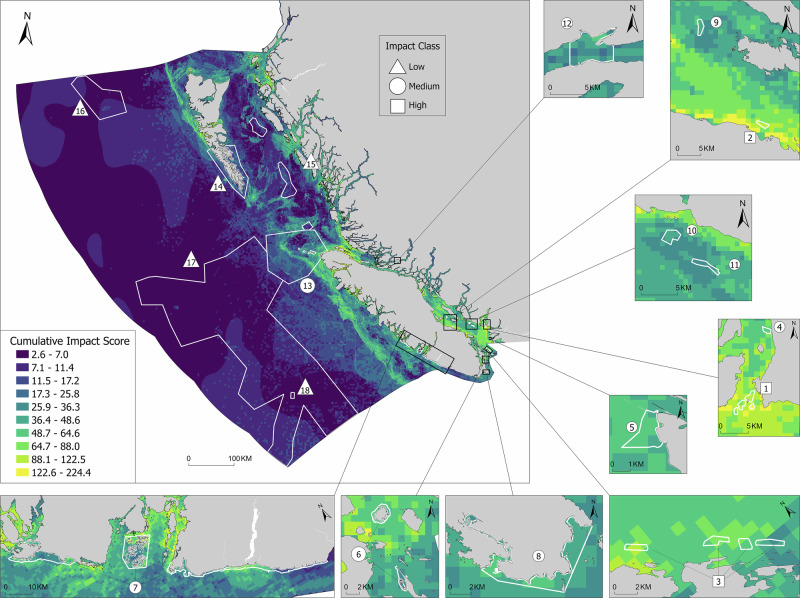
CI scores for existing marine conservation areas (MCAs; *n* = 18) in Pacific Canada. Numbers rank MCAs from highest mean CI score (#1) to lowest mean CI score (#18), and symbols classify mean CI in each MCA as Low, Medium, or High based on Jenks natural breaks for CI scores across the entire region. See Fig. 5 for corresponding names of each MCA and the bioregions in which they are located

**Fig. 5:**
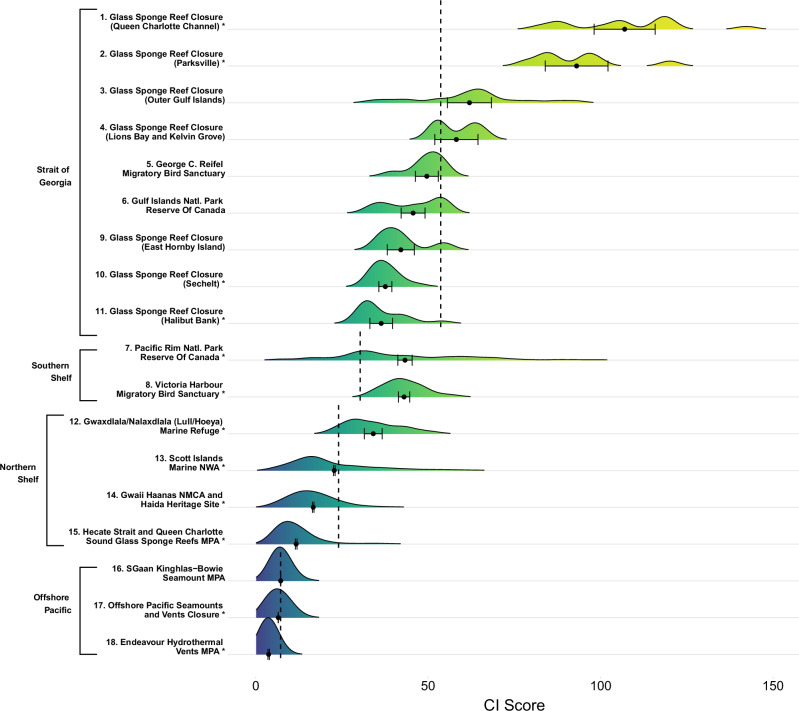
Distribution of CI scores for existing MCAs (*n* = 18) in four bioregions in Pacific Canada. Black circles represent the mean CI score (±95% confidence intervals) within each MCA. Numbers rank MCAs from highest mean CI score (#1) to lowest mean CI score (#18) across the region. Asterisks indicate that the mean CI score in an MCA is significantly different (*p* < 0.05) from the corresponding mean bioregional CI score, inclusive of existing MCAs (dashed lines), according to ANOVA

A wide range of impacts resulting from multiple human activities and stressors are prevalent across the existing MCAs on both the Atlantic and Pacific coasts of Canada (Figs. 2–5). In the Atlantic (Scotian Shelf bioregion), the majority of MCAs were in the low (seven sites) or medium impact classes (six sites), and only three MCAs were in the high impact class (Fig. 2). In the Pacific, over half of the MCAs in the existing marine conservation network were in the medium impact class (ten sites), while five MCAs were in the low impact class and three MCAs were in the high impact class (Fig. 4).

Fifty percent of the existing MCAs in the Scotian Shelf had significantly higher mean CI scores than the overall regional mean, while 38% of the existing MCAs had significantly lower mean CI scores (Fig. 3). We found a similar bi-modal trend for the Pacific MCAs. Compared to the bioregional mean, 28% of the existing Pacific MCAs had significantly higher mean CI scores while 39% had significantly lower mean CI scores (Fig. 5). However, on both coasts, there was a large distribution of CI scores within each MCA (Figs. 3 and 5).

**Fig. 6:**
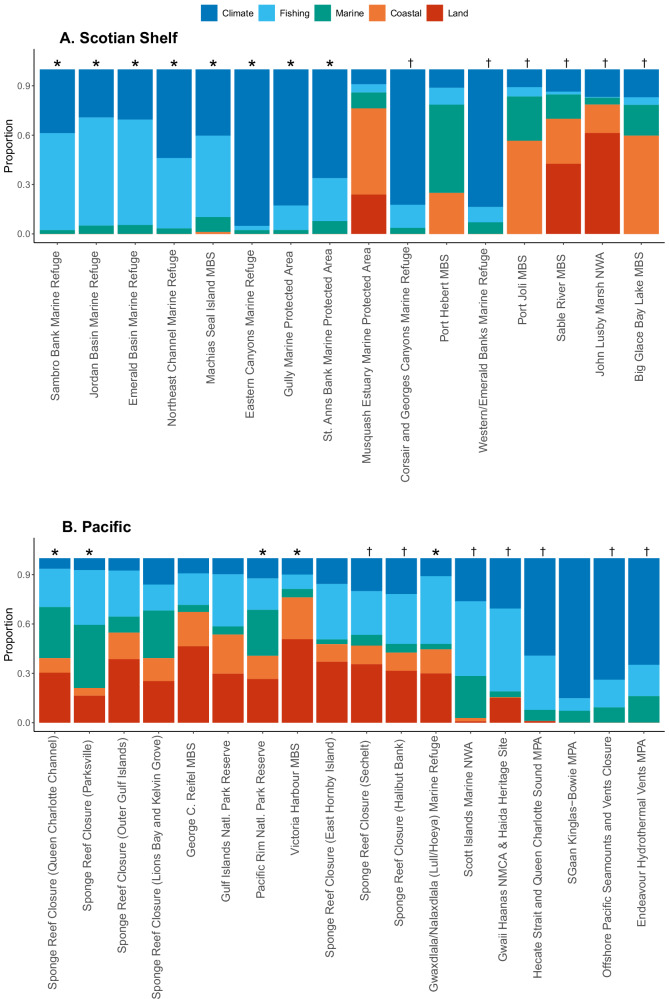
Proportional contribution of each of the five sectors to the mean CI score for each existing MCA in Scotian Shelf (**A**) and Pacific region (**B**). MCAs are ranked from highest mean CI score (left) to lowest (right). Asterisks indicate MCAs whose mean CI score is significantly higher (*p* < 0.05) than bioregional mean CI score, and daggers indicate MCAs whose mean CI score is significantly lower (*p* < 0.05) than bioregional mean CI score according to ANOVA

The sectors contributing to high CI scores in existing MCAs differed between regions. In the Scotian Shelf, climate change stressors and commercial fishing were consistently the largest contributors to CI scores in MCAs, particularly in those MCAs in medium or high impact classes (Fig. 6A). The four MCAs with the lowest CI scores on the Scotian Shelf were primarily influenced by coastal and land-based stressors. In contrast, the Pacific MCAs with the lowest CI scores were offshore sites mostly impacted by climate change and commercial fishing (Fig. 6B). Most Pacific MCAs were influenced by a large range of stressors across all five sectors, with coastal and land-based stressors strongly contributing to cumulative impacts in those Pacific MCAs with the highest mean CI scores (Fig. 6B). In the Scotian Shelf, CI scores in five (out of 16) existing MCAs were primarily (>50% contribution to mean CI score) driven by a combination of land-based stressors (occurring in the watershed) and coastal-based stressors (occurring on land within close proximity to coastline). In the Pacific region, CI scores in four (out of 18) existing MCAs were primarily driven by a combination of land- and coastal-based stressors. Climate change stressors were the primary driver of CI scores in six (out of 16) existing MCAs in the Scotian Shelf, and four (out of 18) existing MCAs in the Pacific.

**Fig. 7:**
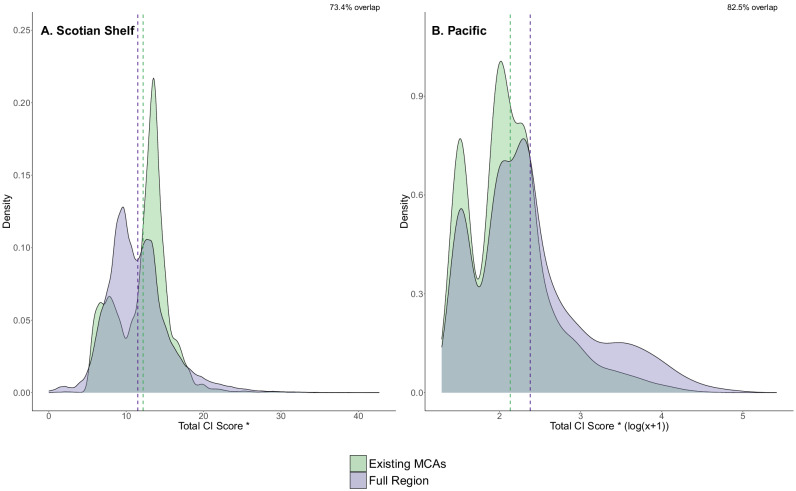
Density plots of total CI scores inside the existing MCAs (green) and in the full region (purple) for the Scotian Shelf (**A**) and Pacific (**B**). Note that the Pacific scores are presented on a log(x + 1) scale. Mean CI scores are indicated as vertical dashed lines in the respective colors. Asterisks indicate significant difference (*p* < 0.05) between mean total CI scores in existing MCAs vs full region, inclusive of MCAs, according to ANOVA

When evaluating the distribution of cumulative impacts in existing MCAs we found that overall existing MCAs in both the Scotian Shelf and Pacific had similar distributions of total CI scores compared to each respective full region. The existing MCAs in the Pacific were more characteristic of total CI scores across the region with 82.5% overlap of frequency distributions, while CI scores in the Scotian Shelf existing MCAs had 73.4% overlap with CI score distribution across the full region (Fig. 7). Existing MCAs in both the Pacific and Scotian Shelf represented the distribution of CI scores from commercial fishing activity well (83.3% and 78.6% overlap, respectively), although areas with the highest impacts from fishing activity tended to fall outside of these MCAs, reflecting design priorities to avoid important fishing grounds (Figures S1 and S2). The overlap of distributions between existing MCAs and the full regions for climate change CI scores differed between the two coasts, with 83.5% overlap in the Pacific and only 61.5% overlap in the Scotian Shelf (Figures S1 and S2). Climate change had the lowest overlap of frequency distributions in the Scotian Shelf, with impacts from climate change stressors higher inside the MCAs compared to the full region (Fig. S1). The distribution of CI scores from marine-based activities in the existing Scotian Shelf MCAs was most similar to the full region (84.8% overlap), while marine-based activities had the lowest percent overlap in the Pacific region (53.9%), with marine-based CI scores lower in the existing MCAs compared to the full region (Figs. S1 and S2).

### Cumulative Impact Protection Potential Across Proposed Marine Conservation Networks

**Fig. 8:**
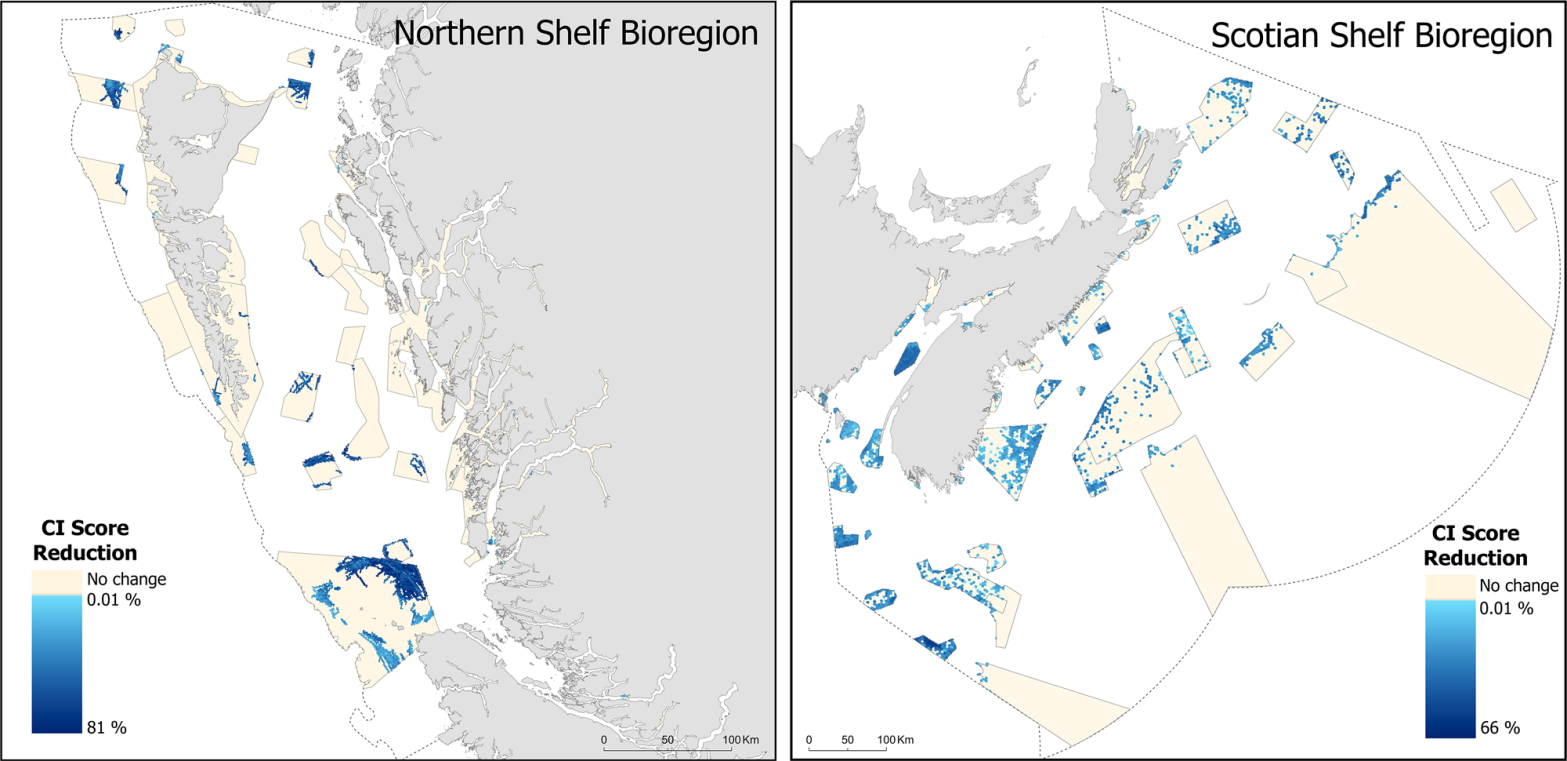
Maps of proposed marine conservation networks (including both existing and proposed MCAs) for Northern Shelf and Scotian Shelf bioregions with estimated percent CI score reduction (per 1 km^2^ cells) from scenario analysis. Cells in MCAs with no change are shown in yellow. Mean percent change per MCA are summarized in Tables S3 (Scotian Shelf) and S4 (Northern Shelf)

Scenario analyses were conducted in the Scotian Shelf and Northern Shelf bioregions to assess how cumulative impacts could be reduced through the implementation of the CMPS in each bioregion’s proposed marine conservation network. Overall, there was a predicted 7.2% mean decrease in CI scores across the 56 proposed and existing MCAs in the Scotian Shelf and 1.9% mean decrease in CI scores across the 37 proposed and existing MCAs in the Northern Shelf (Fig. 8). However, the magnitude of decline in mean CI scores for individual MCAs within the conservation networks varied widely. In the Scotian Shelf bioregion, decline in CI scores following removal of prohibited activities ranged from 0 to 36.4% (Table S3), while in the Northern Shelf bioregion the predicted decline ranged from 0 to 17.2% (Table S4). The CI scores in several MCAs (7 out of 56 in Scotian Shelf bioregion and 16 out of 37 in Northern Shelf bioregion) did not change, as they are located in areas where the prohibited activities from the CMPS would be unlikely to occur and, therefore, there was no spatial overlap (Tables S3 and S4). In the Scotian Shelf, six of the MCAs with no projected CI score change were nearshore sites and one was located offshore past the shelf break (Cold Seeps; Table S3). In the Northern Shelf, all 16 MCAs with no projected CI score change were located in nearshore areas (Table S4).

The scenario analysis also revealed high spatial variability in the distribution of projected CI score declines within each MCA at the 1 km^2^ CI map scale (Fig. 8). Even among MCAs with high protection potential, the projected decline was unevenly distributed, with more pronounced declines concentrated near the edges of the MCAs. At the finer CI map scale, projected CI score decline following removal of prohibited activities ranged from 0 to 66% in the Scotian Shelf bioregion and 0–86% in the Northern Shelf bioregion.

**Fig. 9:**
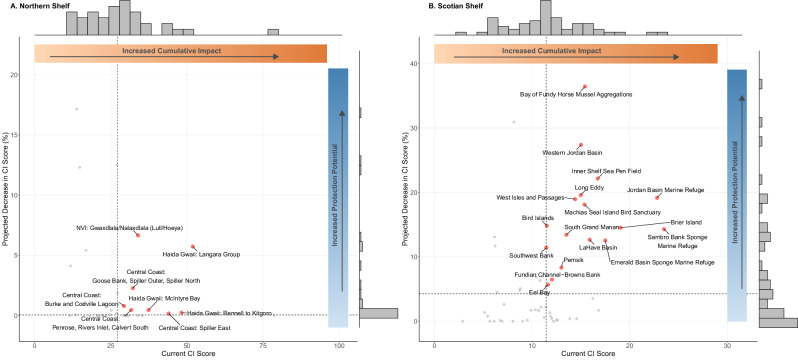
Relationship between current CI score and projected decrease in CI score from scenario analysis for existing and proposed MCAs in the Northern Shelf (**A**) and Scotian Shelf (**B**) bioregional proposed conservation networks. Horizontal and vertical dashed lines indicate median values. MCAs with high protection potential (i.e., those in the top right quadrant with both current CI score and projected decrease in CI score above the median values) are highlighted in red. All other MCAs in the proposed conservation networks are in gray. Gray bar plots are histograms of current CI scores (top) and projected decrease in CI scores (right; Tables S3 and S4). Note: x- and y-axis scales differ between bioregions

Sites with high protection potential were defined as those where both *current CI scores* and *projected declines in CI scores* exceed the respective regional medians. We identified 17 MCAs in the Scotian Shelf and 8 MCAs in the Northern Shelf proposed marine conservation networks as having high potential to reduce cumulative impacts on marine ecosystems following the removal of prohibited activities (bottom contact fishing gear, disposal at sea, and dredging) under the CMPS (Fig. 9; upper right corner). In the Northern Shelf bioregion the three MCAs with the highest protection potential were ‘North Vancouver Island: Gwa̲xdlala/Nala̲xdlala (Lull/Hoeya)’, ‘Haida Gwaii: Langara’, and ‘Central Coast: Goose Bank, Spiller Outer, and Spiller North’ (Fig. 9A). The three MCAs with the highest protection potential in the Scotian Shelf bioregion were the ‘Bay of Fundy Horse Mussel Aggregations’, ‘Western Jordan Basin’ and ‘Inner Shelf Sea Pen Field’ (Fig. 9B).

## Discussion

### Cumulative Impacts Affect MCAs

Cumulative impacts from human activities and stressors are projected to more than double across global marine ecosystems in the next two decades (Halpern et al. 2025). Accounting for these pressures in marine conservation planning is imperative to the success of expanding global conservation efforts, a priority underscored by the UN Global Biodiversity Framework, which calls for broader action to address threats to biodiversity (CBD 2022). Our use of data-driven tools like bioregional CIMs and scenario analyses to quantify impacts and estimate protection potential provides a model for how CIM can be incorporated into decision-making for marine conservation networks. Across both Canadian coasts, our results show that no MCA is immune to cumulative impacts, underscoring continued pressures on biodiversity and ecosystems. As Canada progresses towards its conservation commitments, CIM can provide a timely tool to explicitly integrate threat mitigation directly into the network design and evaluation, guiding both prioritization and ongoing adaptive responses.

Within existing MCAs, a wide range of CI scores were observed across both regions, encompassing low to high impact classes. This observation likely reflects trade-off analyses during the conservation planning process to minimize socioeconomic impacts (Beaty et al. 2024) and stress-mitigation choices to balance recovery opportunities in high-impact areas and preservation of undistributed impacts in low-impact areas (King et al. 2021; Martone et al. 2021). However, given these MCAs are under spatial protection measures, one may expect that cumulative impacts would be lower within MCA boundaries than outside. Potential reasons for not observing the expected lower CI scores within all MCAs could be a result of a limited range of prohibited activities for some MCAs, a mismatch in the spatial resolution of the stressor data and MCA boundaries, diffuse pressures from outside MCAs (e.g., climate change stressors), temporal data mismatch, or that many of the MCAs are vertically zoned where core protection surrounds benthic features and does not restrict pelagic activities. Indeed, existing MCAs on both coasts show CI score distributions that resemble the gradient of CI scores observed in the surrounding unprotected areas in their planning regions, indicating that these networks of existing measures are not biased towards any part of the cumulative impact spectrum.

High CI scores can originate from both proximate and distant stressors. Within MCAs, such scores may reflect larger-scale drivers beyond the boundaries and thus do not necessarily indicate inadequacy of applied regulations. The CIM model includes climate change stressors along with marine-, coastal-, and land-based activities with varying footprints that can affect marine ecosystems. Therefore, even fully protected marine areas can have high CI scores driven by distantly originating stressors, such as sedimentation from forestry activities influencing coastal habitats via river runoff (Saunders et al. 2017). Our results showed that land-based stressors strongly contribute to CI scores in about one-quarter of existing MCAs on both coasts. However, because these activities fall outside the scope of marine conservation planning, they are often not managed within MCAs, resulting in lower cumulative impact reduction in some existing MCAs. Further, diffuse stressors such as persistent pollutants and ocean warming associated with climate change can affect even the most remote MCAs (Bruno et al. 2018; Hamilton et al. 2023; Lewis et al. 2023). While all MCAs on both coasts are influenced by climate change stressors to some extent, we found that 38% of existing MCAs in the Scotian Shelf and 22% of existing MCAs in the Pacific had CI scores that were highly influenced by climate change stressors. Thus, even with management of marine-based stressors, these MCAs will continue to experience relatively high cumulative impacts. Knowledge of the extent and type of interactions between local (e.g., fishing, aquaculture, etc.) and global (e.g., climate change) stressors can be used to guide effective management decisions, and local-scale activities that overlap with areas of high climate change impacts will require careful management (Brown et al. 2013; Brown et al. 2014). Considering the cumulative impacts of climate change stressors in marine conservation planning can aid in the implementation of proactive conservation measures (Bryndum-Bucholz et al. 2023; Rubidge et al. 2024; Keen et al. 2024). For example, buffering against climate-driven impacts to ecosystem resilience by managing additional local stressors (Wilson et al. 2020; Bryndum-Bucholz et al. 2022). Marine conservation is a key tenet of ecosystem-based management, a broader integrative approach that seeks to balance ecological integrity with sustainable human use (Long et al. 2015). The goal of conservation within a MSP context is not to eliminate human activity, but to strategically reduce pressures on ocean ecosystems while balancing ecological, economic, and social objectives. Embedding conservation planning within an ecosystem-based management framework that integrates across sectors and supports a thriving blue economy, will be essential to meeting biodiversity and ecosystem preservation commitments under the GBF, while also advancing sustainable ocean use (Halpern et al. 2010; Tam et al. 2024; Bundy et al. 2025). Our results provide valuable insights that highlight MCAs particularly at risk from climate change or land-based stressors to support integrated ecosystem-based management.

High CI scores associated with several existing MCAs on both coasts (e.g., Sambro Bank and Emerald Basin Marine Refuges in the Scotian Shelf, glass sponge reef closures in the Strait of Georgia, and Gwa̲xdlala/Nala̲xdlala (Lull/Hoeya) Marine Refuge in the Northern Shelf) are also caused in part by temporal misalignment between the data used to represent activities in the CIMs and the timing of MCA establishment. For example, the data used to represent commercial fishing activity in the Scotian Shelf CIM was compiled from catch records between 2009-2018, while the fishing regulations applied within Sambro Bank and Emerald Basin Marine Refuges were implemented in 2013. The Gwa̲xdlala/Nala̲xdlala (Lull/Hoeya) Marine Refuge in the Northern Shelf bioregion was established in 2023, but the data used to represent commercial fishing activity in this area of the Pacific CIM was from 2007 – 2016. Therefore, the CI scores reflect benthic fishing activities that occurred prior to MCA regulations being enacted. Current recommendations to update CIMs every five to ten years (Halpern et al. 2015) can improve these estimates by incorporating human activity data that better aligns with MCA establishment timelines. Nevertheless, the current analysis provides a valuable baseline for tracking future changes as more MCAs become established, and can also be beneficial in monitoring management effectiveness within MCAs going forward.

Our results highlight a divide in the types of human activities and stressors driving high cumulative impacts on each coast. Commercial fishing and climate change are the main contributors to high cumulative impacts in Scotian Shelf MCAs, while Pacific MCAs with high cumulative impacts were influenced by activities from all five sectors, with particularly high contributions from land- and coastal-based activities, along with marine activities outside of commercial fishing. This trend reflects an inherent difference between how humans utilize the ocean on each coast. Commercial fishing has been a foundational industry in the Scotian Shelf for centuries and remains the dominant ocean industry in the region (DFO 2024b). Other marine-based activities like aquaculture and shipping are a larger part of ocean industry in the Pacific, and land-based impacts resulting from high population density and forestry are also prevalent. Thus, while coastal MCAs on the Scotian Shelf have generally low CI scores, the coastal MCAs in the Pacific bioregions have among the highest CI scores across the region. This disparity is an important consideration in designing MCAs on either coast. The design should account for which impacts can be addressed through site-specific regulations and which require management in adjacent areas or jurisdictions (e.g., provincial vs. federal governments).

### Protection Potential of Marine Conservation Networks

Across both regions, conservation networks are being developed as composites of existing measures and draft areas that, when implemented, would serve to meet regional conservation objectives. These measures offer an opportunity both for biodiversity conservation and to help mitigate local and regional human pressures on marine ecosystems. Understanding how these draft areas overlap with cumulative impacts, and how regulatory tools (e.g., prohibitions under the CMPS) could be applied, can illustrate how MCAs and regional networks would contribute to cumulative impact mitigation.

We quantified the protection potential for the Scotian Shelf and Northern Shelf bioregions by modeling the removal of prohibited activities outlined in the CMPS and subsequent decline in CI scores. Identifying MCAs with high potential for impact reduction can be a valuable decision support tool for conservation planners (Furlan et al. 2020; Holness et al. 2022) in addition to a new integrative metric for quantifying the conservation value associated with site and network establishment. We identified 17 MCAs in the Scotian Shelf and 8 MCAs in the Northern Shelf proposed conservation networks with strong potential for cumulative impact reduction following removal of prohibited stressors in the scenario analysis. Among those with high protection potential were Jordan Basin Marine Refuge in the Scotian Shelf and Gwa̲xdlala/Nala̲xdlala (Lull/Hoeya) Marine Refuge in the Northern Shelf which have both been established as MCAs in 2016 and 2023, respectively. The selection and designation of these MCAs in the planning process aligns well with the high protection potential we identified in the scenario analysis. Future CIM updates will likely reveal significant reductions in CI scores for these MCAs.

Despite a similar distribution of high, medium, and low impact classes in the existing MCAs on both coasts, the scenario analysis predicted a larger decline in cumulative impacts across the Scotian Shelf’s proposed marine conservation network (7.2%) compared to the Northern Shelf’s network (1.9%). This difference can largely be attributed to a lower occurrence of bottom-contact commercial fisheries in many of the Northern Shelf bioregion’s existing and proposed MCAs, which are instead largely affected by activities and stressors not addressed by the CMPS. Still, large declines are projected in the Northern Shelf bioregion, especially when changes are assessed at the scale of individual MCAs, as opposed to the entire network. For example, 15 (out of 37) MCAs in the Northern Shelf bioregion’s proposed conservation network contain areas (i.e., 1 km^2^ CI map grid cells) where CI scores are projected to decrease by more than 50%. These large, localized CI score decreases at the 1 km^2^ grid-cell scale are primarily driven by the removal of bottom-trawl fishing gear. In the Scotian Shelf bioregion’s proposed conservation network, seven (out of 56) MCAs contain areas with greater than 50% projected CI score decrease. The large, localized CI score declines in the Scotian Shelf are driven by a combination of bottom-trawl and dredge boat fishing gear removal. This finding indicates that while many existing and proposed MCAs in the Northern Shelf bioregion contain small areas with large projected CI score declines, average decline is generally greater in the Scotian Shelf bioregion MCAs. The 1 km² resolution of the CI maps enables detailed MCA assessments, revealing localized declines within many MCAs following management action, even when the mean CI score decline across the entire MCA is smaller.

In the scenario analysis, we removed activities and associated stressors defined under the CMPS. The CMPS specifies baseline prohibitions for federal MCAs established after April 25, 2019, whereas MCAs established before this time are not automatically subject to these prohibitions (DFO 2023). Consequently, this analysis represents a best-case baseline for federal MCAs established after 2019, rather than a universal baseline for all MCAs. In addition, each bioregion’s conservation network includes MCAs classified into distinct types based on their management objectives, legal designation, and lead federal agency. For example, MPAs and Marine Refuges that are established by Fisheries and Oceans Canada often focus on protecting specific ecological features, habitats, or species using fisheries closures, while Marine National Wildlife Areas (mNWA) are established by Environment and Climate Change Canada to protect critical habitat for wildlife with a focus on migratory bird species (ECCC 2025a). The mNWAs prohibit the activities defined in the CMPS, but like other types of MCAs, additional restrictions can be included. For example, the Scott Islands mNWA in the Northern Shelf bioregion has several additional restrictions in place, including the restriction of anchoring vessels and human access within close proximity to the coastline, among others (ECCC 2025b). The scenario analysis methodology could be applied using MCA-specific activity restrictions to better support management decisions regarding individual MCAs, potentially revealing even greater protection potential in some MCAs.

### Uncertainties and Assumptions

This CIM framework is conceptually straightforward but data-intensive and inherits uncertainties similar to those described by Halpern and Fujita (2013). Outputs depend on data availability and quality. We included only those datasets available at a comparable scale and do not account for unreported or illegal activities (e.g., poaching or fishing infractions). We aimed to select activities with similar temporal ranges and spatial resolution, but alignment was not always possible. Temporal mismatches mainly reflect differing establishment timelines among MCAs, so regional assessments may reflect pre- and post-establishment periods. Spatial resolution mismatch may also occur in some input datasets where some input activity data were coarser than the native resolution of the analysis (e.g., commercial fishing data from Scotian Shelf was at a 10 km^2^ resolution) and therefore should be interpreted cautiously at this finer resolution. We did not include MCAs smaller than 1 km^2^ in the current analysis for this reason. Higher resolution analyses may be needed for smaller MCAs. Caution should be exercised in design decisions when impacts are assessed at a spatial resolution finer than the CIM as scale mismatches can distort the magnitude and distribution of projected effects.

Finally, the original CIM outputs used in these analyses were generated based on the vulnerability of general habitat classes (e.g., soft bottom shelf, eelgrass meadows, etc.) to stressors, as opposed to specific conservation priorities. Conservation priorities are often unique to each MCA and include ecosystem components to be protected, such as kelp beds or hydrothermal vent habitats. Some of the MCA conservation priorities in both regions’ networks are consistent with the habitat classes used to generate CI scores in the CIMs, but not all. Calculating CI scores using the vulnerability of specific conservation priorities to different stressors would result in scores that are more relevant to site-based management and conservation planning. This could be applied in the future with the development of vulnerability scores for the specific conservation priorities of each MCA, following methods such as Murray et al. (2024). However, at broader network scales, CIMs remain a valuable planning tool, offering an integrated view of human pressures and helping to identify areas for protecting relatively unimpacted habitats, as well as areas where conservation actions could support ecological restoration (e.g., Gilby et al. 2021).

### Conclusions

Beyond its illustration value, CIM can be used to prioritise activities and stressors with high impact for management or mitigation action. For example, Mach et al. (2017) evaluated cumulative impacts to MPAs in California and identified activities under local management control that contribute to cumulative impacts in the context of global climate change. CIM can also be used to identify monitoring indicators which can be used to guide monitoring for the effects of external and diffuse stressors, as well as to stratify monitoring efforts across a range of cumulative impacts as a proxy for levels of disturbance. As an explicit part of MSP, CI scores could be used as a cost layer in Marxan analyses to identify or compare planning scenarios for MCA networks.

Biodiversity loss and declining ecosystem function threaten marine ecosystems and the services that underpin a thriving blue economy globally (Worm et al. 2006). As the United Nations Global Biodiversity Framework calls for a balanced approach to the use and protection of ecosystems, tools that translate multiple pressures into actionable priorities will be essential. Cumulative impact mapping provides an integrative spatial lens that can help identify where conservation action will have the greatest effect. Our findings highlight a range of cumulative impacts within existing Canadian MCAs and demonstrate how CIM can help prioritize high-impact activities and stressors for management or mitigation within a conservation planning context across two proposed Canadian marine conservation networks. It also reveals how to strengthen existing conservation measures where recovery is feasible. Our scenario analyses indicate that restricting key activities within these proposed networks could yield meaningful impact reductions, enabling networks to stay aligned with Global Biodiversity Framework goals while addressing cumulative pressures through spatial planning. CIM can contribute to conservation objectives not only by informing coordinated planning that supports the establishment of new sites, but also by guiding improvements to existing measures through enhanced stressor mitigation and adaptive management as pressures evolve (Stanley et al. 2025). Embedding CIM within MSP can help to align spatial conservation measures with the broader objective of managing human activities and the ecosystems that support them. For MCAs, regular CIM updates could provide an important context for stakeholder engagement helping to bolster adaptive management and conservation outcomes, and ultimately support a resilient blue economy.

## Supplementary information

Supplementary information

## Data Availability

Data used in this study is publicly available at the following links: https://open.canada.ca/data/en/dataset/8a08603a-a60c-4ca5-aab8-614ed14bc68f and https://open.canada.ca/data/en/dataset/37b59b8b-1c1c-4869-802f-c09571cc984b.
