# Supplementary material for: A Coast-to-Coast Assessment of Cumulative Impacts and Protection Potential in Canadian Marine Conservation Networks
Source: Environ Manage. 2026 Apr 16;76(5):158. doi: 10.1007/s00267-026-02427-y (PMC13086886; doi:10.1007/s00267-026-02427-y)
Supplement: Supplementary file 1 — Supplementary information [file 267_2026_2427_MOESM1_ESM.docx]

Supplementary Material

A coast-to-coast assessment of cumulative impacts and protection potential in Canadian marine conservation networks

Grace E.P. Murphy^a^, Selina Agbayani^b^, Jocelyn C. Nelson^c^, Emily M. Rubidge^b^, Ryan R.E. Stanley^a^, Cathryn C. Murray^b^, and Noreen E. Kelly^a^

^a^ Bedford Institute of Oceanography, Fisheries & Oceans Canada, 1 Challenger Drive, Dartmouth, NS B2Y 4A2, Canada

^b^ Institute of Ocean Sciences, Fisheries & Oceans Canada, 9860 West Saanich Road, Sidney, BC, V8L 4B2, Canada

^c^ Pacific Biological Station, Fisheries & Oceans Canada, 5190 Hammond Bay Road, Nanaimo, BC, V9T 6N7, Canada

Table S1. Human activity layers included in the Scotian Shelf (n = 45) and Pacific (n = 46) cumulative impact maps and their associated sectors. See Murphy et al. (2024) and Agbayani et al. (2024) for a description of the data used to create each layer and the specific stressor for which each activity serves as a proxy.

| Sector | Scotian Shelf Activities | Pacific Activities |
| --- | --- | --- |
| Climate change | 1. Acidification 2. Bottom temperature change 3. Surface temperature change | 1. Surface temperature change |
| Commercial Fishing | 1. Fishing vessel traffic 2. Angling rod and reel 3. Danish or Scottish seine 4. Dredge boat 5. Gillnet 6. Hand line baited 7. Harpoon and spear 8. Longline groundfish 9. Longline pelagic 10. Pots (all types) 11. Purse tuck seine 12. Shrimp trawl 13. Trap net 14. Troller lines 15. Bottom otter trawl | 1. Fishing vessel traffic 2. Crab trap 3. Prawn trap 4. Sablefish trap 5. Geoduck dive 6. Sea cucumber dive 7. Green urchin dive 8. Red urchin dive 9. Groundfish bottom trawl 10. Groundfish midwater trawl 11. Shrimp trawl 12. Scallop trawl 13. Herring roe gillnet 14. Herring roe seine 15. Halibut hook and line 16. Lingcod hook and line 17. Rockfish hook and line 18. Salmon gillnet 19. Salmon seine 20. Salmon trawl 21. Euphasiid seine 22. Tuna hook and line |
| Marine-based | 1. Aquaculture: finfish 2. Aquaculture: shellfish 3. Benthic structures 4. Commercial vessel traffic 5. Contaminated sites 6. Disposal at sea 7. Invasive species 8. Lost fishing gear 9. Recreational boat traffic 10. Unexploded explosive ordinances | 1. Aquaculture: finfish 2. Aquaculture: shellfish 3. Anchor scouring 4. Commercial vessel traffic 5. Dredging 6. Disposal at sea 7. Recreational boat traffic 8. Recreational fishing: groundfish hook and line 9. Recreational fishing: tuna hook and line 10. Recreational fishing: salmon hook and line 11. Recreational fishing: shellfish trap |
| Coastal-based | 1. Coastal industrial tenures 2. Coastal power plants 3. Coastal trash 4. Diseases and pathogens 5. Energy infrastructure: LNG 6. Hardened shoreline 7. Light pollution 8. Marinas/boat launches/harbors 9. Marine tourism operators 10. Marine tourism: trails, beaches, campgrounds 11. Ports 12. Pulp and paper mills | 1. Commercial anchorages 2. Log booms 3. Marinas 4. Ports |
| Land-based | 1. Agriculture land cover 2. Historical gold mines 3. Impervious surface cover 4. Nutrient loading 5. Population density | 1. Agriculture 2. Mining 3. Industrial tenures 4. Forestry 5. Human settlements 6. Pulp and paper mills 7. Roads 8. Sewage outfalls |

Table S2. Habitats included in the Scotian Shelf and Pacific cumulative impact maps. See Murphy et al. (2024) and Agbayani et al. (2024) for a description of the data used to create each layer.

| Habitat zone | Scotian Shelf | Pacific |
| --- | --- | --- |
| Intertidal (0-2m) | 1. Beach 2. Tidal flat 3. Rocky intertidal 4. Saltmarsh 5. Algal zone | 1. Soft intertidal 2. Mixed intertidal 3. Hard intertidal 4. Undefined intertidal 5. Eelgrass 6. Kelp |
| Nearshore (< 30m) | 1. Eelgrass 2. Kelp 3. Nearshore soft bottom 4. Nearshore mixed bottom 5. Nearshore hard bottom | 1. Soft bottom shallow 2. Mixed bottom shallow 3. Hard bottom shallow 4. Undefined bottom shallow |
| Offshore (30 – 200m) | 1. Horse mussel bioherm 2. Soft bottom shelf 3. Mixed bottom shelf 4. Hard bottom shelf | 1. Sponge reefs 2. Soft bottom shelf 3. Mixed bottom shelf 4. Hard bottom shelf 5. Undefined shelf |
| Deep (>200m) | 1. Soft bottom bathyal 2. Mixed bottom bathyal 3. Hard bottom bathyal 4. Deep biogenic 5. Canyon | 1. Soft bottom slope 2. Mixed bottom slope 3. Hard bottom slope 4. Undefined slope 5. Soft bottom deep 6. Mixed bottom deep 7. Hard bottom deep 8. Undefined deep |
| Varying depths |  | 1. Soft canyons 2. Mixed canyons 3. Hard canyons 4. Undefined canyons 5. Hard hill 6. Undefined hill 7. Hard knoll 8. Undefined knoll 9. Soft seamount 10. Mixed seamount 11. Hard seamount 12. Undefined seamount |
| Pelagic | 1. Shallow pelagic 2. Deep pelagic | 1. Shallow pelagic 2. Deep pelagic |


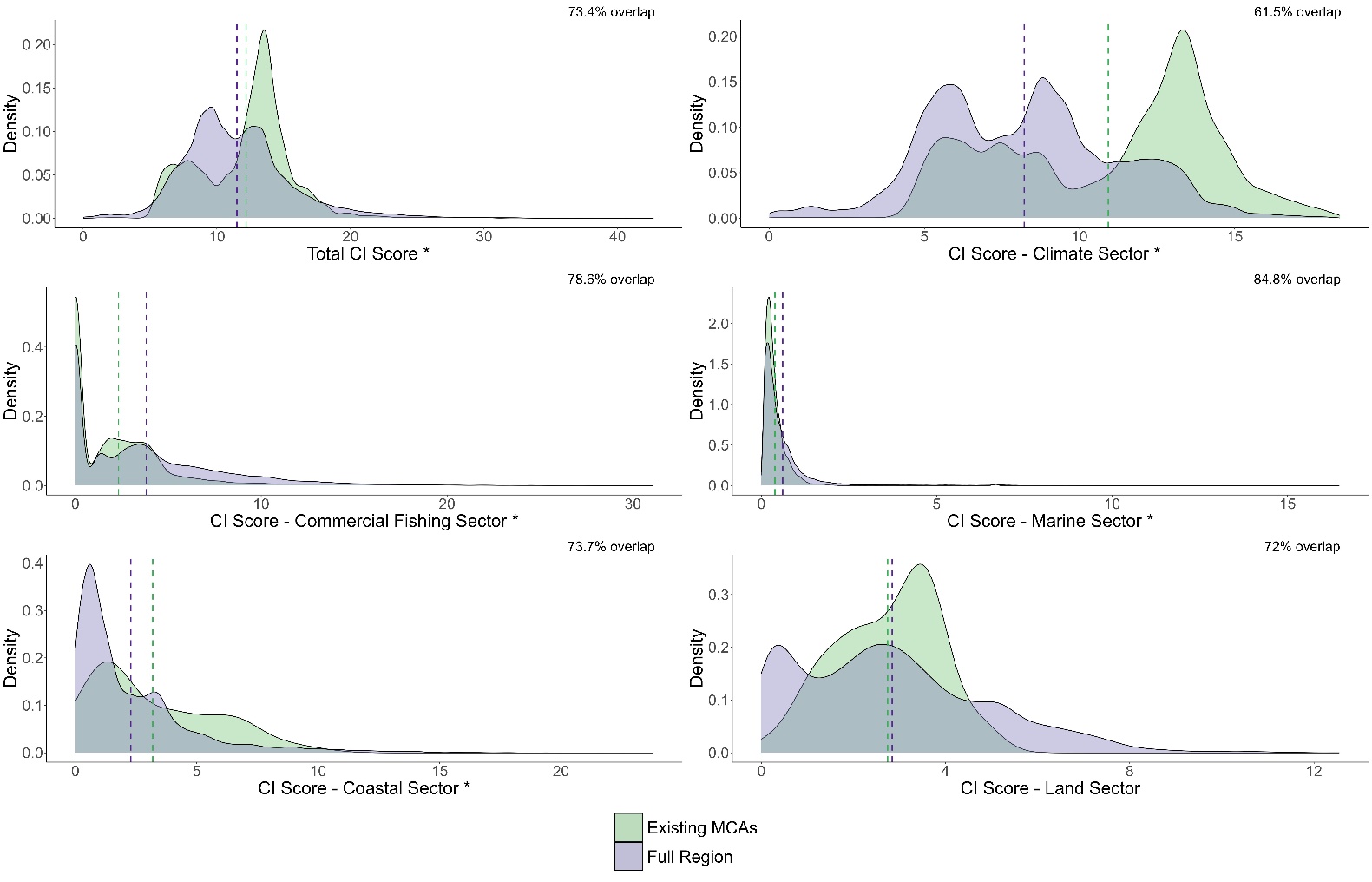


Figure S1. Density plots of total CI scores and sector-specific CI scores for the Scotian Shelf bioregion inside the existing MCAs (green) and in the full region (purple). Mean CI scores are indicated as vertical dashed lines in the respective colours. Asterisks next to sector names indicate significant difference (p < 0.05) between mean CI scores in existing MCAs vs full region.


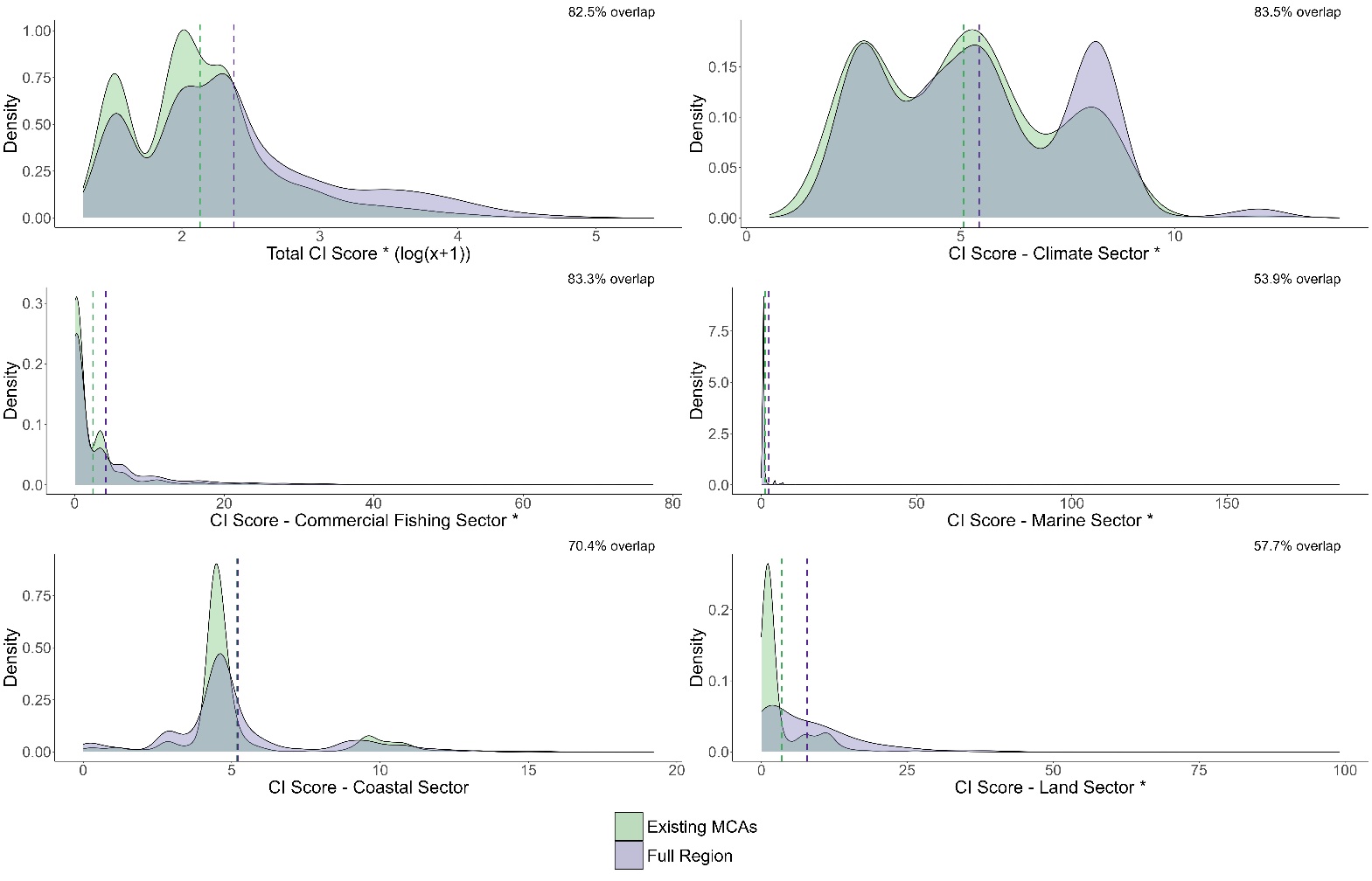


Figure S2. Density plots of total CI scores and sector-specific CI scores for the Pacific region inside the existing MCAs (green) and in the full region (purple). Mean CI scores are indicated as vertical dashed lines in the respective colours. Asterisks next to sector names indicate significant difference (p < 0.05) between mean CI scores in existing MCAs vs full region.

Table S3. Mean percent CI score reduction estimated from scenario analysis in MCAs included in the Scotian Shelf bioregion proposed conservation network.

| *MCA Name* | *CI Score % Reduction* |
| --- | --- |
| Bay of Fundy Horse Mussel Aggregations | 36.4 |
| Georges Bank | 30.9 |
| Western Jordan Basin | 27.4 |
| Inner Shelf Sea Pen Field | 22.1 |
| Long Eddy | 19.6 |
| Jordan Basin Marine Refuge | 19.2 |
| West Isles and Passages | 18.9 |
| Machias Seal Island Bird Sanctuary | 18.1 |
| Bird Islands | 14.8 |
| Brier Island | 14.5 |
| Sambro Bank Marine Refuge | 14.3 |
| South Grand Manan | 13.4 |
| Salmon Rivers | 13.1 |
| LaHave Basin | 12.6 |
| Emerald Basin Sponge Marine Refuge | 12.5 |
| Eastern Shoal | 11.7 |
| Southwest Bank | 11.4 |
| Pemsik | 8.3 |
| Fleur de Lis Coast | 7.0 |
| Scotian Gulf | 6.9 |
| Fundian Channel-Browns Bank | 6.5 |
| Canso Bank and Channels | 6.3 |
| Eel Bay | 5.7 |
| Logan Canyon | 5.4 |
| Misaine Bank and Laurentian Channel | 5.0 |
| Southern Bight | 4.7 |
| Sable Island Bank | 4.5 |
| McNutts Island | 4.3 |
| St. Anns Bank Marine Protected Area | 4.1 |
| MacNamaras Islands | 3.5 |
| Western/Emerald Banks Marine Refuge | 2.6 |
| Canso Ledges - Sugar Harbour Islands | 2.2 |
| Eastern Shore Islands | 2.2 |
| Chebogue | 1.8 |
| Country Island | 1.7 |
| Northeast Channel Coral Marine Refuge | 1.7 |
| Musquash Estuary Marine Protected Area | 1.7 |
| Cobequid Bay | 1.4 |
| Martinique Beach & Musquodoboit Harbour | 1.3 |
| Chignecto Bay | 0.8 |
| LaHave Islands | 0.8 |
| Ingonish | 0.6 |
| Aspy Bay | 0.4 |
| Sambro Ledges - Prospect | 0.4 |
| Eastern Canyons Marine Refuge | 0.4 |
| Gully Marine Protected Area | 0.2 |
| Central Scotian Slope, Rise and Abyss | 0.2 |
| Corsair and Georges Canyons Marine Refuge | 0.2 |
| Bras d'Or Lake | 0.1 |
| Big Glace Bay Lake Migratory Bird Sanctuary | 0.0 |
| Cold Seeps | 0.0 |
| John Lusby Marsh National Wildlife Area | 0.0 |
| Port Hebert Bird Sanctuary | 0.0 |
| Port Joli Bird Sanctuary | 0.0 |
| Sable River Bird Sanctuary | 0.0 |
| St. Mary's River and Estuary | 0.0 |

Table S4. Mean percent CI score reduction estimated from scenario analysis in MCAs included in the Northern Shelf bioregion proposed conservation network.

| *MCA Name* | *CI Score % Reduction* |
| --- | --- |
| Haida Gwaii: Dogfish Bank | 17.2 |
| North Vancouver Island: Scott Islands mNWA | 12.5 |
| Haida Gwaii: Queen Charlotte Sound | 12.3 |
| North Vancouver Island: Gwa̲xdlala/Nala̲xdlala (Lull/Hoeya) | 6.7 |
| Haida Gwaii: Langara Group | 5.7 |
| Haida Gwaii: Offshore | 5.4 |
| Hectate Strait/Queen Charlotte Sound Glass Sponge Reefs MPA | 4.1 |
| Central Coast: Goose Bank, Spiller Outer, and Spiller North | 2.3 |
| Central Coast: Hakai Pass | 1.9 |
| Gwaii Haanas NMCA | 1.0 |
| Central Coast: Burke and Codville Lagoon | 0.8 |
| Central Coast: Penrose, Rivers Inlet, and Calvert South | 0.5 |
| Haida Gwaii: McIntyre Bay | 0.5 |
| North Coast: Kitkatla Inlet | 0.4 |
| Haida Gwaii: Rennell to Kitgoro | 0.2 |
| Central Coast: Hakai South | 0.2 |
| Central Coast: Spiller East | 0.2 |
| North Coast: Queen Charlotte Sound | 0.1 |
| North Coast: Caamano Sound | 0.1 |
| Central Coast: Kitasu-Laredo | 0.1 |
| Haida Gwaii: Five Mile Group | 0.0 |
| Central Coast: Bentick Arm | 0.0 |
| Central Coast: Dean, Cascade, Labouchere, and Kimsquit | 0.0 |
| Central Coast: Kitasu-Price and Kitasu-Aristazabal | 0.0 |
| Central Coast: Kitasu-Jackson and Millbanke | 0.0 |
| Central Coast: Green Inlet and Khutze | 0.0 |
| Central Coast: Kynoch Group | 0.0 |
| Haida Gwaii: Masset Inlet | 0.0 |
| Haida Gwaii: Skidegate Inlet | 0.0 |
| Haida Gwaii: Unknown mNWA | 0.0 |
| Haida Gwaii: Naden Group | 0.0 |
| Haida Gwaii: Beresford to Hippa | 0.0 |
| Haida Gwaii: Englefield to Tasu | 0.0 |
| North Coast: Banks Aristazabal | 0.0 |
| North Coast: Kitkiata and Kishkosh Inlets | 0.0 |
| North Coast: Kiltuish Butedale | 0.0 |
| North Vancouver Island: Heydon and Loughborough | 0.0 |
